# The Relationship between Vitamin C and Periodontal Diseases: A Systematic Review

**DOI:** 10.3390/ijerph16142472

**Published:** 2019-07-11

**Authors:** Akio Tada, Hiroko Miura

**Affiliations:** 1 Department of Health Science, Hyogo University, 2301 Shinzaike Hiraoka-cho, Kakogawa, Hyogo 675-0195, Japan; 2 Department of International Health and Collaboration, National Institute of Public Health, 2-3-6, Minami, Wako, Saitama 351-0197, Japan

**Keywords:** Vitamin C, ascorbic acid, periodontal disease, periodontitis, gingivitis

## Abstract

Vitamin C is important for preventing and slowing the progression of many diseases. There is significant evidence linking periodontal disease and vitamin C. We aimed to systematically review the studies addressing the relationship between vitamin C and periodontal disease, and the preventive ability of vitamin C against periodontal disease. Electric searches were performed using PubMed, EMBASE, Cochrane Library, and Web of Science. Studies addressing the relationships between periodontal disease and vitamin C in adults aged over 18 years were included. Quality assessment was done using the Critical Appraisal Skills Program guideline and GRADE-CERQual. There were 716 articles that were retrieved and 14 articles (seven cross-sectional studies, two case-control studies, two cohort studies, and three randomized controlled trials (RCT)) were selected after reviewing all of the articles. The vitamin C intake and blood levels were negatively related to periodontal disease in all seven cross-sectional studies. The subjects who suffer from periodontitis presented a lower vitamin C intake and lower blood-vitamin C levels than the subjects without periodontal disease in the two case-control studies. The patients with a lower dietary intake or lower blood level of vitamin C showed a greater progression of periodontal disease than the controls. The intervention using vitamin C administration improved gingival bleeding in gingivitis, but not in periodontitis. Alveolar bone absorption was also not improved. The present systematic review suggested that vitamin C contributes to a reduced risk of periodontal disease.

## 1. Introduction

### 1.1. Background

A large number of people, approximately 90% of the global population, suffer from periodontal diseases [1,2]. Progressed periodontitis destroys the alveolar bone that supports teeth, eventually leading to a loss of teeth. Moreover, periodontitis possibly increases the risk of various other diseases, such as diabetes mellitus type 2 and cardiovascular diseases, and has adverse pregnancy outcomes [3].

Periodontal disease is an inflammatory disease that is initiated by bacterial infection, subsequently progresses via an aberrant host response, and primarily contributes to periodontal tissue destruction [4]. Polymorphonuclear leukocytes (PMNs) are critically involved in biophylaxis against periodontopathogens [5,6]. They induce an antimicrobial response at the site of infection by triggering various intracellular signaling pathways, including reactive oxygen species (ROS) [7]. ROS have a cytotoxic effect on periodontal tissues at higher concentrations [8,9]. Oxidative stress by PMNs may be the primary reason for the damage of periodontal tissue in periodontal disease [10]. Patients with periodontitis demonstrate higher levels of biomarkers that indicate ROS-induced-tissue damage than the controls [11,12,13].

Vitamin C is an important nutrient that exerts a reducing and anti-oxidant effect, scavenges free radicals, and acts as an enzyme cofactor in cells [14,15]. As vitamin C scavenges excessive ROS, this nutrient is considered to be an important dietary oxidant for periodontal health [16]. Vitamin C also plays a crucial role in preventing and slowing the progression of periodontal disease by inducing the differentiation of periodontal ligament progenitor cells [17].

To prevent periodontal disease, evidence addressing nutritionally effective approaches must be generated. In the last two decades, several epidemiological studies have evaluated the association between vitamin C intake and periodontal disease. In addition, the ability of vitamin C to prevent periodontal disease has been analyzed in clinical studies. Vitamin C has been quantified using dietary intake or blood-vitamin C concentrations in these studies. Furthermore, periodontal disease consists of gingivitis and periodontitis, which have different pathologies, and possibly different associations with vitamin C. However, no comprehensive systematic review of the literature has been conducted to date.

### 1.2. Objectives

Therefore, this study aimed to systematically review the relationship between the dietary intake and blood concentration of vitamin C and periodontal disease, as well as the preventive ability that vitamin C exerts against periodontal disease.

## 2. Material and Methods

To minimize bias, we developed a protocol to address the a priori research questions, comprehensive literature search with inclusion and exclusion criteria for studies, screening methods, data abstraction, scientific study quality, and data analysis.

### 2.1. Literature Search

In the present systematic review, the inclusion criteria were defined according to the Preferred Reporting Items for Systematic Reviews and Meta-Analyses (PRISMA) guidelines and the population (P; “human adults”), intervention or exposure (I; “impact of vitamin C on periodontal disease”), comparison (C; “different amounts of vitamin C intake, different concentration of blood vitamin C, or no administration of vitamin C”), and outcome (O; “periodontal disease parameters”) model (PICO model) [18]. Two independent authors (Akio Tada and Hiroko Miura) assessed the eligibility of the studies by screening the titles and abstracts, according to the PICO model. The following PICO question was used: “Does vitamin C associate with periodontal status?”. Furthermore, all of the studies that met the following inclusion criteria: (1) written in English, (2) published between July 1998 and June 2018, (3) investigating the association between periodontal disease and vitamin C, (4) conducted on adult subjects (age ≥18 years), and (5) using quantitative methods of data collection, were included in this review. Epidemiological studies on adults were included.

A literature search was performed using the PubMed, EMBASE, Cochrane Library, and Web of Science databases using the following search items: (“periodontal disease” and “vitamin C”, or “periodontal disease” and “ascorbic acid”).

### 2.2. Quality Assessments

The studies, including cohort studies, case-control studies, and randomized controlled trials (RCTs), that met the inclusion criteria were assessed using the Critical Appraisal Skills Programme (CASP) in terms of the quality of the methodology [19]. The checklist for the cohort studies was partially modified and applied to cross-sectional studies (e.g., questions concerning the follow-up of participants were excluded). Two independent authors (Akio Tada and Hiroko Miura) calculated the strengths and weaknesses of each study using the CASP checklist items, and assigned them a grade of “high”, “moderate”, or “low”. Two independent authors (Akio Tada and Hiroko Miura) resolved any disagreements through consensus.

The GRADE CERQual was used to assess the confidence of the key findings of this systematic review. Two reviewers independently reviewed the findings according to the guidance for GRADE CERQual parameters [20]. CERQual bases this evaluation on the following four criteria: (a) methodological limitations of the included studies supporting a review finding, (b) relevance of included studies to the review question, (c) coherence of the review finding, and (d) adequacy of the data contributing to a review finding.

### 2.3. Data Extraction

The data were extracted from the selected literature by two independent authors (Akio Tada and Hiroko Miura) using a specifically developed data extraction sheet. The disagreements were resolved via consensus after discussion. The relevant data extracted from each study that were rated as eligible included first the author; publication year; setting; type of study; number of subjects; confounding factors; and main findings, including both adjusted odds ratios and 95% confidence intervals (CIs).

## 3. Results

### 3.1. Literature Searches and Study Characteristics

The initial database search yielded 716 relevant studies, and the records were retrieved through a manual search. After the removal of the duplicate records, 240 records were screened. Of these studies, 194 were excluded based on title and abstract screening. The remaining 46 articles were screened by a further analysis.

**Figure 1:**
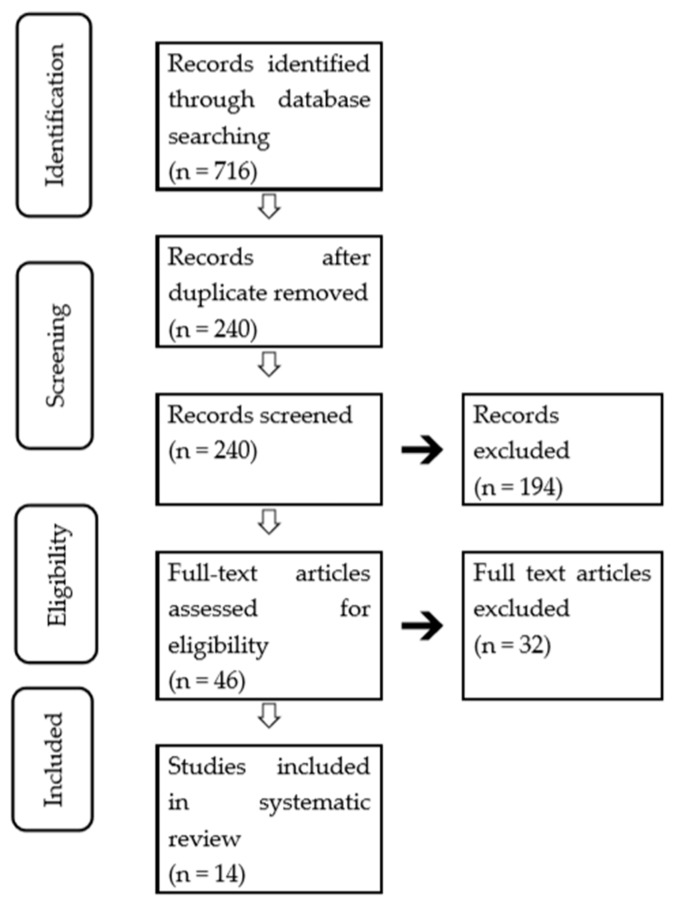
Flow diagram of the literature search.

From these 46 potential relevant articles, 32 did not meet the inclusion criteria. Finally, 14 publications (seven cross-sectional studies [21,22,23,24,25,26,27], two case-control studies [28,29], two cohort studies [30,31]), and four RCTs [32,33,34]) were selected for this systematic review, as shown in the flow chart (Figure 1).

Excluding the RCTs, six of the remaining 11 studies estimated the vitamin C intake from a dietary investigation, and six studies measured the blood vitamin C concentration. One study evaluated vitamin C based on both dietary intake and blood concentrations. The intervention studies compared the status of the periodontal tissues before and after vitamin C administration.

The assessment indications for periodontal disease included the community periodontal index (CPI), pocket depth (PD), attachment loss (AL), bone loss, clinical attachment level (CAL), bleeding on probing (BOP), gingival index (GI), gingivitis severity index (GSI) and sulcus bleeding index (SBI).

### 3.2. Quality Evaluation

The quality evaluation of the studies was performed using CASP, as presented in Table 1, Table 2, Table 3 and Table 4. The strengths and limitations of each study were discussed therein. All of the included studies clearly focused on an issue. The subjects were recruited in an acceptable manner in all of the observational studies (*n* = 11; 100%). All of the studies measured the outcomes in order to minimize bias (*n* = 14; 100%). Regarding dietary exposure, four cross-sectional studies [21,22,23,24], one case-control study [29], and one cohort study [30] assessed the dietary intake. Of these studies, five used dietary recall [21,22,23,24,29]; specifically, two studies used multiple dietary recalls [22,29], and three used a single dietary recall [21,23,24]. As a single 24-h dietary recall could not represent one’s habitual diet [35], multiple 24-h recalls are desirable, and this technique was rated as “satisfactory”. Two studies using multiple dietary recalls were rated as “satisfactory”. One study used food frequency questions (FFQs) that were previously validated [30], and was rated as “satisfactory”. Six studies used serum/plasma vitamin C levels for assessment [28,29,30,31,32,34] and were rated as “satisfactory”.

The incidence and development of periodontal disease are influenced by various factors, including demographic factors, socioeconomic factors, smoking systemic diseases (e.g., diabetes and obesity), and oral health habits [36,37,38,39,40,41]. When analyzing the relationship between the relevant factor(s) and periodontal disease in a regression analysis, these potential confounders must be controlled for in cross-sectional studies and cohort studies. Five studies covered all of these confounders [21,22,23,30,31], and were subsequently rated as “satisfactory”.

The quality of these studies was assessed with CASP according the number of “satisfied” scores, as follows: cohort studies = high (9), moderate (8–10), and low (7 or less); others = high (9), moderate (7–8), and low (6 or less). Six studies were rated “high” and nine were rated as “moderate”.

We analyzed the confidence of our five findings using CERQual. Three findings were assigned a moderate confidence rating, and two were assigned a low confidence rating. A detailed explanation of the confidence ratings is presented in Table 5.

### 3.3. Relationship between Vitamin C Intake/Level to Periodontal Disease in Cross-Sectional Studies

Four studies analyzed the relationship between vitamin C intake and periodontal disease. Two studies used the same subjects (participants in the fourth Korean National Health and Nutrition Examination Survey) [21,23]. Both of the studies set 2/3 as the cut-off score for the CPI. Lee et al. observed that the highest quartile (>132.2 mg/day) of vitamin C intake had a significantly lower CPI score than the lowest quartile (≤47.34 mg/day) among adults aged 19 years and older (Table 6). Park et al. showed that the lower intake group had a significantly greater proportion of CPI (≥3) than the higher intake group, using a cut-off point of the median value of vitamin C intake (81.3 mg/day), among women or nonsmokers aged 19–39 years [23] (Table 6).

Two other large-scale studies have reported that periodontal markers exhibit significant associations with the dietary intake of vitamin C. Luo and colleagues showed that those with an insufficient vitamin C intake had more severe periodontal disease [22] (Table 6). Another study revealed an association between reduced dietary vitamin C and an increased risk for periodontal disease [24] (Table 6).

Three studies have investigated the correlation between clinical attachment loss and serum/plasma vitamin C levels [24,25,26]. Two studies have shown that the extent of attachment loss was negatively correlated to the plasma vitamin C levels among subjects. Amarasena et al. demonstrated that the serum vitamin C concentrations were negatively correlated to clinical attachment loss [26] (Table 6). Chapple et al. demonstrated that the prevalence of severe periodontitis was significantly higher in the subjects with serum vitamin C levels <8.52 mmol/L, compared with subjects with other vitamin C concentrations [25] (Table 6). One study demonstrated that the extent of attachment loss was negatively correlated to the plasma vitamin C levels among the subjects [27] (Table 6).

### 3.4. Relationship between Vitamin C Intake/Level and Periodontal Disease in Case-Control Studies

The serum vitamin C levels were compared between the patients with periodontal disease and the controls in two case-control studies [28,29]. Both of the studies demonstrated that periodontitis patients have significantly lower serum vitamin C levels compared with the controls (Table 7).

### 3.5. Relationship between Vitamin C Intake/Level and Periodontal Disease in Cohort Studies

Two cohort studies were included in the systematic review [30,31]. The subjects of these studies were the same people living in Japan. Populations with a higher dietary intake or higher serum levels of vitamin C exhibited slower periodontal disease progression (Table 8).

### 3.6. Improvement in Periodontal Status by Vitamin C in RCTs

Four studies have analyzed the preventive effects of vitamin C administration intervention on periodontal status. One study investigated the effect of the local (dentifrice containing ascorbic acid) administration of vitamin C on periodontal disease [32] (Table 9). Dentifrice presented an improvement in reducing gingivitis [32]. Grapefruit consumption improved the sulcus bleed index in patients with chronic periodontitis [34]. Two additional studies involving clinical trials investigated the additional effect of vitamin C combined with non-surgical periodontal disease treatment. Gokhale et al. presented that non-surgical treatment combined with vitamin C supplementation exhibited a significant reduction in the SBI of patients with gingivitis [33] (Table 9). In contrast, vitamin C does not exhibit an additional effect on the improvement in the clinical measures of periodontitis [33,34] (Table 9).

## 4. Discussion

Literature review articles addressing the association between periodontitis and various nutrients, including vitamin C, have previously been published [42,43,44]. To the best of our knowledge, this is the first systematic review that considers the findings and critically appraises the quality of the studies addressing the relationship between vitamin C and periodontal disease.

### 4.1. Quality Assessment of the Studies

Several studies reviewed in this review article used dietary intake data to assess vitamin C intake. Three studies used a single dietary recall [24,26,27]. As these studies used large-scale samples from national surveys, obtaining data on multiple dietary recalls might have been difficult. Nevertheless, the studies employed multiple dietary recalls, validated FFQ, or objective evaluations by measuring the blood vitamin C level. The evaluation of vitamin C from these articles is considered reliable.

Three of the eight cross-sectional studies and both of the cohort studies were also scored “satisfactory” for controlling for confounders. Moreover, although three studies did not control for all of the potential confounders, these studies controlled for almost all of the potential confounders when assessing the relationship between the vitamin C levels and periodontitis [25,26,27], thereby increasing the reliability of the current review’s findings.

The confidence levels of the findings ranged from moderate to high, based on the CERQual assessments. The main evaluation criteria for downgrading were the methodological limitations and adequacy of the studies. Concerns about the methodological limitations included a sufficient adjustment for the confounding factors. A downgrade in adequacy occurred as a result of the concern about the richness of the data contributing to the review findings. Nonetheless, the concerns found in coherence and relevance were limited, and no study showed contradictory data in the relationship between vitamin C and periodontal disease, which provides higher confidence to the findings concerning this relationship.

### 4.2. Impact of Vitamin C on Periodontal Status

All of the cross-sectional studies demonstrated that periodontal disease was significantly associated with the vitamin C dietary intake or blood vitamin C level. The subjects with periodontitis exhibited higher blood vitamin C levels than the control in two of the case control studies. These findings suggest that the vitamin C obtained from the diet is transferred to the periodontal tissue via blood circulation, thus decreasing the risk of periodontal disease. However, a reverse causation could be deduced from the associations presented in the cross-sectional studies. Thus, it is possible that the associations may represent the effects of periodontal disease on vitamin C intake as a result of decreased mastication rather that the effect of vitamin C intake on periodontitis. Longitudinal studies are needed in order to elucidate and verify this association.

A longitudinal relationship between vitamin C and periodontal disease was presented in two cohort studies in this review [30,31]. These results support the mechanism that the vitamin C obtained from diet reduces the inflammatory reaction in periodontal disease. However, given that these two cohort studies used the same population, further research using cohort studies is necessary in order to reinforce the reliability of the relationship.

Two of the three RCTs demonstrated improvements in periodontal indices (GI, SBI, or PD) [32,33], such as an improvement in gingival condition, caused by vitamin C administration. The administration of vitamin C alone or with non-surgical treatment has shown effects for improving gingival indices, SBI, and GI. Vitamin C has a powerful anti-oxidative effect in living organisms, particularly at the intracellular level [45], and this is thought to decrease the oxidative stress generated in gingivitis. Additionally, vitamin C reduced the cytotoxic and apoptotic activity of *Porphyromonas gingivalis* in human periodontal ligament cells and human gingival fibroblasts [46,47], which may have contributed to these effects. However, the reduction in the SBI score by vitamin C has not been observed in periodontitis patients [33,34]. Vitamin C also reduced gingival bleeding in gingivitis lesions, but not in periodontitis lesions. It has been speculated that when the inflammatory reaction extends from the gingiva to other periodontal tissues, including the alveolar bone, some factors that inhibit the effect of vitamin C as an anti-oxidant may be generated.

Vitamin C administration did not demonstrate an improvement in the pocket depth [33]. Another study also reported the inefficacy of vitamin C administration in improving the pocket depth and attachment level [48]. A reduction of the pocket depth requires the regeneration of the alveolar bone. Although vitamin C induces the in vitro osteogenic differentiation of periodontal ligament progenitor cells [17], there has been no report that vitamin C triggers the bone regeneration in vivo, which is a likely explanation for the lack of pocket depth reduction after vitamin C administration.

Nevertheless, vitamin C administration has been observed to improve periodontal disease to some extent in the intervention studies reviewed. It could be considered that the findings obtained from the articles reviewed in this study were sufficient to demonstrate the preventive ability of vitamin C with regard to periodontal disease.

There remains a possibility that periodontopathic pathogens lower the blood vitamin C level by biodegradation. However, one study reported that no significant changes in the serum ascorbic acid levels could be observed in patients with moderate to severe periodontitis after treatment with scaling and root planning [49]. This study suggests that the concentration of vitamin C in the blood influences the periodontal status, but not vice versa, indicating that a change in periodontal health does not influence the blood vitamin C level.

Some articles included in this review analyzed the effects of other vitamins having an anti-oxidative effect, such as vitamin A and vitamin E, on periodontal disease [25,26,32,33]. Among the anti-oxidant vitamins, only vitamin C showed a consistent association with periodontal disease in these studies. It can be considered feasible and meaningful to analyze the association of vitamin C with periodontal disease

### 4.3. Influence of Other Factors on the Association between Vitamin C and Periodontal Disease

A close link has been established between diabetes and periodontitis, and diabetes has a negative influence on the healing of oral surgical wounds [50]. The associations among vitamin C, periodontitis, and diabetes are of interest. The key articles in this review showed some evidence on the association between vitamin C and periodontal diseases in patients with diabetes. In Gokhale’s study, vitamin C exhibited an additional effect on the improvement of gingival bleeding in periodontitis patients with diabetes [33]. Lee et al. demonstrated that patients with pre-diabetes and diabetes exhibited a stronger association between vitamin C and periodontitis than those with normal blood sugar levels [21]. The patients with diabetes also showed low ascorbate levels [51]. This could be because glucose inhibits the transportation of vitamin C to the cells, and because of the stimulation of the hexose monophosphate shunt by vitamin C [52,53]. In diabetic patients, vitamin C is considered to work less effectively. However, unlike in these studies, the key articles showed that vitamin C that is administered or taken in via food is likely to work effectively. Vitamin C also enhances the immune function by supporting various cellular functions by of both the innate and adaptive immune systems [54], which may influence the periodontal status of diabetic patients. It is speculated that there might be more complex interactions among vitamin C, diabetes, and periodontal disease. Further intervention studies are warranted in order to elucidate this mechanism.

Park et al. stated that the association between vitamin C and periodontitis was significant in non-smokers, but not significant in smokers [23] In contrast, Nishida et al. showed that vitamin C intake was significantly associated with periodontitis in current smokers and past smokers, but not in non-smokers [24]. Although differences were found in the periodontal markers, vitamin C, and the adjusted confounding factors between these two studies, the differences in their results cannot be sufficiently explained by the differences in these factors. Vitamin C is known to reduce the oxidative stress caused by nicotine [55]. Some studies consistently demonstrated that smokers have lower vitamin C levels in the plasma and leukocytes than non-smokers, probably because of the increased oxidative stress [56]. Complicated interactions may exist between the anti-oxidative effect of vitamin C and the oxidative stress effect induced by tobacco on the periodontal tissue. Further studies are needed in order to elucidate this perplexing mechanism(s).

The possibility of associations between Interferon (IFN)-γ in cervical fluid and periodontal disease was suggested [57]. Vitamin C has been reported to increase the IFN level in virus-infected human cells [58]. It is of interest to examine whether vitamin C influences the expression of IFNs.

### 4.4. Limitations

First, the periodontal disease indicators used in the reviewed studies were diverse, and this precluded us in making comparisons between the results of the studies. The evaluation for periodontal disease differs according to the indicator used, positively affecting the relationship between periodontal disease and vitamin C. For example, CPI has a risk of underestimation due to partial mouth recording [59]. The possibility of a fluctuation of results as a result of inconsistencies in the evaluation of the indicators attenuates the reliability of this study.

Secondly, smoking and diabetes are factors that exacerbate periodontal disease and negatively influence the anti-inflammatory function of vitamin C. Some interactions may exist between vitamin C and the molecules released from tobacco or those highly expressed in diabetes. However, the evidence about this concern that is available from the articles included in this review is perplexing, and the current study provides insufficient evidence addressing this concern.

Third, the number of articles for the review is small. Nevertheless, the articles reviewed in this study found a relationship between vitamin C and periodontal disease, which validates this relationship.

### 4.5. Future Direction

The use of different indicators for periodontal disease makes it impossible to compare the strength of associations between vitamin C and periodontal disease obtained from multiple studies, because of a lack of easily identifiable standards. The quantitative assessment of the periodontal status and the treatment effect of periodontal disease requires a unification of indicators.

The possibility that smoking and diabetes pathology influences the preventive ability of vitamin C against periodontal disease was suggested by the studies analyzing the association of these factors with vitamin C [51,55,56]. Further population-based research addressing this point may be of help in order to identify the comprehensive measures for preventing periodontal disease.

Vitamin C can easily be consumed through a variety of foods. There is a report that the consumption of grapefruit, which is rich in vitamin C, improved the sulcus bleed index in patients with chronic periodontitis [60]. Collaboration between oral health professionals and dieticians in public health sectors is expected to be more effective when promoting oral health in community dwellings. Future studies should aim to identify the merits of collaboration between nutritionists and dental health professionals.

The antiseptic effect of the topical application of chlorhexidine has been examined and demonstrated [61]. It is of interest to investigate the effect of vitamin C combined with chlorhexidine for the prevention of and lowering the progression of periodontal disease.

## 5. Conclusions

This review provided an overview and appraisal of studies analyzing the relationship of vitamin C to periodontal disease. It highlights the effects of vitamin C on the prevention of incidence and the development of periodontal disease. Further studies addressing the use of unified periodontal indicators and an elucidation of the implications of other factors should be performed so as to increase knowledge of the relationship between vitamin C and periodontal disease.

## Figures and Tables

**Table 1:** The results of the critical appraisal assessments for cross-sectional studies.

Reference	1	2	3	4	5a	5b	6	7	Quality Evaluation
Lee [21]	*1*	*1*	*0*	*1*	*1*	*1*	*1*	*1*	*Moderate*
Luo [22]	*1*	*1*	*1*	*1*	*1*	*1*	*1*	*1*	*High*
Park [23]	*1*	*1*	*0*	*1*	*1*	*1*	*1*	*1*	*Moderate*
Nishida [24]	*1*	*1*	*0*	*1*	*0*	*0*	*1*	*1*	*Moderate*
Chapple [25]	*1*	*1*	*1*	*1*	*0*	*0*	*1*	*1*	*Moderate*
Amarasena [26]	*1*	*1*	*1*	*1*	*0*	*0*	*1*	*1*	*Moderate*
Amaliya [27]	*1*	*1*	*1*	*1*	*0*	*0*	*1*	*1*	*Moderate*

*1—*satisfied; *0*—not satisfied. 1—Clearly focused issue; 2—Adequate recruitment method; 3—Adequate measurement of exposure; 4—Adequate measurement of outcome; 5a—Identification of all of the important confounding factors; 5b—Adequate study design accounting for the confounding factors; 6—Application of the results to the population of a local area; 7—Agreement with other available evidence.

**Table 2:** The results of the critical appraisal assessments for case-control studies.

Reference	1	2	3	4	5a	5b	6	7	Quality Evaluation
Kuzmanova [28]	*1*	*1*	*1*	*1*	*1*	*1*	*1*	*1*	*High*
Staudte [29]	*1*	*1*	*1*	*1*	*1*	*0*	*1*	*1*	*moderate*

*1—*satisfied; *0*—not satisfied. 1—Clearly focused issue; 2—Adequate recruitment method; 3—Adequate selection of controls; 4—Adequate measurement of exposure; 5a—Equal treatment of the groups; 5b—Adequate study design accounting for the confounding factors; 6—application of the results to the population of a local area; 7—Agreement with other available evidence.

**Table 3:** The results of the critical appraisal assessments for cohort studies.

Reference	1	2	3	4	5a	5b	6a	6b	7	8	Quality Evaluation
Iwasaki [30]	*1*	*1*	*1*	*1*	*1*	*1*	*0*	*1*	*1*	*1*	*Moderate*
Iwasaki [31]	*1*	*1*	*1*	*1*	*1*	*1*	*0*	*1*	*1*	*1*	*Moderate*

*1*—satisfied; *0*—not satisfied. 1–5b—Same as cross-sectional study; 6a—Completion of follow-up; 6b—Sufficient length of follow up; 7—Application of the results to the population of a local area; 8—Agreement with other available evidence.

**Table 4:** The results of the critical appraisal assessments for randomized controlled trials (RCTs).

Reference	1	2	3	4	5	6	7	8	9	Quality Evaluation
Shimabukuro [32]	*1*	*1*	*1*	*1*	*1*	*1*	*1*	*1*	*1*	*High*
Gokhale [33]	*1*	*1*	*1*	*1*	*1*	*1*	*1*	*1*	*1*	*High*
Abou [34]	*1*	*1*	*1*	*1*	*1*	*1*	*1*	*1*	*1*	*High*

*1—*satisfied; *0*—not satisfied. 1—Clearly focused issue; 2—Randomized assignment of patients; 3—Proper selection of patients; 4—Blinded experiment; 5—Similarity of the groups at the beginning of the trial; 6—Equal treatment of the groups; 7—Application of the results in the context; 8—Consideration of clinically important outcomes; 9—Benefits outweigh harms and costs.

**Table 5:** The results of CERQual grading.

Key Finding	Studies Supporting Key Finding	Methodological limitations	Relevance	Coherence	Adequacy	Overall Assessment of Confidence	Explanation of Judgement
① Adults with a lower dietary vitamin C intake have a higher incidence and severity, and more progressions of periodontal disease than those with a higher dietary vitamin C intake	[21,22,23,24,28,30]	Minor methodological concerns in 1/6 studies, with adjustment for confounding factors	No or very minor concerns about relevance	No or very minor concerns about coherence	Minor concerns about adequacy, as information lacked richness (1/6)	High	Finding graded as high because of only minor concerns about methodological quality and adequacy of contributing papers
② Adults with lower blood vitamin C levels have a higher incidence and severity, and more progressions of periodontal disease than those with a higher dietary vitamin C level	[25,26,27,28,29,31]	Moderate methodological concerns in 3/6 studies, with adjustment for confounding factors, and in one study with sampling	No or very minor concerns about relevance	No or very minor concerns about coherence	Moderate concerns about adequacy, as information lacked richness (2/5)	Moderate	Finding downgraded because of concerns about methodological quality and adequacy of contributing papers
③ Administration of vitamin C improving periodontal disease	[32,33,34]	No or very minor methodological concerns	Minor concerns about relevance on the specification of intervention (1/3)	Minor concerns about coherence, given that the effect is limited on gingivitis	Moderate concerns about adequacy of data, given the small number of studies	Moderate	Finding downgraded because of relevance, coherence, and adequacy concerns of contributing papers

**Table 6:** Summary of cross-sectional studies on the relationship between vitamin C and periodontal disease.

Reference	Study Sample	Measurement of Vitamin C	Measurement of Periodontal Status	Control of Confounding Factors ^a^	Key Results
Lee et al. [21]	10,930 individuals (≥19 years; Korea)	A 24-h dietary record (adequate/inadequate vitamin C intake)	CPI score; periodontitis; CPI = 3 or 4	1, 2, 3, 4, and 5	Lowest intake (<47.3 mg/day) vs highest intake (≥132.2 mg/day); adjusted odds ratio (aOR) = 1.28 (95% confidence interval (CI) = 1.10–1.50)
Park et al. [23]	2049 individuals (19–39 years; Korea)	Complete one-day 24-h recall interviews	CPI score; periodontitis; CPI = 3 or 4	1, 2, 3, and 4	Lower intake (<81.3 mg/day) vs higher intake (≥81.3 mg/day); aOR = 1.66 (95% CI = 1.04–2.64) for women; aOR = 1.49 (95% CI = 1.04–2.14) for nonsmokers
Luo et al. [22]	6415 individuals (≥30 years; U.S.)	24-h recall interviews	PD; AL; increased severity	1, 2, 3, 4, and 5	Vitamin C intake ≤20.65 mg/day vs ≥112.91/da7; aOR = 1.401 (95% CI = 1.12–1.74)
Nishida et al. [24]	12,419 individuals (20 years and over; U.S.)	24-h dietary record	Clinical attachment level; periodontal disease ≥1.5	1 and 3	Vitamin C intake (<0–29 mg/day) vs (>180 mg/day); aOR = 1.30
Chapple et al. [25]	11,895 individuals (≥20 years; U.S.)	Serum vitamin C and anti-oxidant concentration	AL; PD; severe periodontitis: ≥2; mesiobuccal sites with AL ≥5 mm and ≥1; mesiobuccal sites with PD ≥4 mm	1, 2, 3, and 5	Serum vitamin C concentration: highest (>70.41 mmol/L) vs lowest (<8.52 mmol/L); aOR = 0.53 (95% CI = 0.42–0.68)
Amarasena et al. [26]	413 individuals (70 years and older; Japan)	Serum vitamin C	AL	1, 3, 4, and 5	Serum vitamin C level-attachment loss: coefficient = −0.04 (95% CI = −0.06 to −0.005)
Amaliya et al. [27]	123 individuals (33–43 years; Indonesia)	Plasma vitamin C	AL	1, 2, 3, and 4	Plasma vitamin C- attachment loss; coefficient = −0.199

^a^ The following variables were adjusted in the analyses: 1—demographic factors; 2—socioeconomic factors; 3—smoking/alcohol; 4—flossing/brushing; 5—diabetes, hypercholesterolemia, hypertension, and obesity.

**Table 7:** Summary of the case-control studies on the relationship between of vitamin C and periodontal disease.

Reference	Study Sample	Measurement of Vitamin C	Measurement of Periodontal Status	Key Results
Kuzmanova et al. [28]	21 patients with periodontitis and 21 controls (≥19 years, Dutch)	Vitamin C plasma level	Bone loss periodontitis >1/3 of the root length	Plasma vitamin C level: periodontitis patients < controls (*p* = 0.03)
Staudte et al. [29]	42 patients with periodontitis (mean age 43.7 years) and 38 controls (mean age 40.5 years; Germany)	Seven-day food record; vitamin C plasma level	PD; chronic periodontitis: having ≥5 teeth with periodontal sites exhibiting PDs ≥3.5mm	Plasma vitamin C level: periodontitis patients < controls (*p* < 0.05); dietary intake of vitamin C: patients < controls (*p* < 0.05)

The following variables were adjusted in the analyses: 1—demographic factors; 2—socioeconomic factors; 3—smoking/alcohol; 4—flossing/brushing; 5—diabetes, hypercholesterolemia, hypertension, and obesity.

**Table 8:** Summary of cohort studies on the relationship between of vitamin C and periodontal disease.

Reference	Study Sample	Measurement of Vitamin C	Measurement of Periodontal Status	Control of Confounding Factors ^a^	Key Results
Iwasaki et al. [30]	264 individuals (77 years; Japan)	Food frequency questions	Number of teeth having an AL of 3 mm or greater regression (8 years prospective)	1, 2, 3, 4, and 5	Lowest vitamin C intake (reference) vs. middle: 0.76 (0.60–0.97) vs. highest: 0.72 (0.56–0.93)
Iwasaki et al. [31]	264 individuals (72 years; Japan)	Serum vitamin C	Number of teeth having AL of 3 mm or greater regression (2 years retrospective)	1, 2, 3, 4, and 5	Highest vitamin C level (reference) vs. middle: 1.12 (1.01–1.26) vs. lowest: 1.30 (1.16–1.47)

^a^ The following variables were adjusted in the analyses: 1—demographic factors; 2—socioeconomic factors; 3—smoking/alcohol; 4—flossing/brushing; 5—diabetes, hypercholesterolemia, hypertension, and obesity.

**Table 9:** Summary of RCT studies in the improvement of periodontal status by vitamin C.

References	Study Sample	Intervention	Measurement of Periodontal Status	Key Results
Abou et al. [34]	30 individuals with chronic periodontitis (Syria)	Non-surgical periodontal therapy and vitamin C administration	PD; CAL; BOP; GI	Vitamin C did not offer an additional effect to non-surgical periodontal therapy on the improvement in clinical measures
Shimabukuro et al. [32]	300 individuals with gingivitis (Japan)	Dentifrice containing L-ascorbic acid 2-phosphate magnesium salt	GSI	GI test group: from 1.22 ± 0.03 to 0.73 ± 0.03; GI control: from 1.16 ± 0.03 to 0.84 ± 0.03; GSI test group: from 1.09 ± 0.04 to 0.69 ± 0.03; GSI control: from 1.13 ± 0.04 to 0.78 ± 0.03
Gokhale et al. [33]	120 individuals (30–60 years; India)	Non-surgical periodontal therapy (scaling and root planning: SRP) and vitamin C administration	SBI; PD	SBI—mean of differences (scores at baseline − scores after two weeks); SRP + vitamin C: 0.56 ± 0.26; SRP: 0.28 ± 0.12; PI and PD were not unaffected
